# Diagnosis, Treatment, and Management for Chronic Coronary Syndrome: A Systematic Review of Clinical Practice Guidelines and Consensus Statements

**DOI:** 10.1155/2023/9504108

**Published:** 2023-12-18

**Authors:** Tianyue Jing, Yu Wang, Yukun Li, Liangyu Cui, Xingfang Liu, Dasheng Liu, Cong Ren, Tong Yin, Zhiwei Zhao, Jiaheng Wang, Xuejie Han, Liying Wang

**Affiliations:** ^1^Institute of Basic Research in Clinical Medicine, China Academy of Chinese Medical Sciences, Beijing, China; ^2^Research Department, Swiss University of Traditional Chinese Medicine, Bad Zurzach, Switzerland; ^3^Shaanxi University of Chinese Medicine, Xianyang, Shaanxi, China

## Abstract

**Objectives:**

Management of chronic coronary syndrome (CCS) encompasses a broad spectrum of practices, posing considerable complexity and variability. While guidelines have been established to augment the management quality of CCS, notable disparities persist across their recommendations. This study strives to scrutinize, compare, and reconcile these guideline recommendations pertaining to the diagnosis, treatment, and management of CCS patients. Our goal is to align these recommendations with contemporary clinical practices, thus laying a robust foundation for their pragmatic application in clinical settings.

**Methods:**

A comprehensive systematic search was conducted across multiple databases, including PubMed, EMBASE, China National Knowledge Infrastructure, Wanfang Database, China Science and Technology Journal Database, Chinese Biomedical Literature Service System, Chinese Science and Technology Periodical Database, and Chinese Biological Medicine Database. The timeframe for this search spanned from their inception up to May 30, 2022, aiming to collate all published guidelines relevant to CCS. Subsequently, two independent reviewers undertook the task of appraising the quality of these guidelines by utilizing the Appraisal of Guidelines for Research and Evaluation II instrument.

**Results:**

The search yielded a total of 10,699 citations. Following a thorough evaluation, fourteen clinical practice guidelines and four consensus statements, each offering specific recommendations for CCS, were selected. The quality of these guidelines showcased a broad spectrum of variation. The domain of “presentation clarity” received the highest accolades, while “applicability” languished at the lower end of the scoring spectrum. On average, the guidelines attained a quality score denoting sufficiency. Furthermore, recommendations across different guidelines for the diagnosis, treatment, and management of CCS displayed a striking level of divergence.

**Conclusion:**

The landscape of published CCS guidelines is marked by extensive variations in scope, quality, and recommendations. Hence, there is a compelling need for collaborative efforts amongst multidisciplinary professionals to forge comprehensive, higher-quality evidence-based guidelines; such a concerted approach is paramount to enhance treatment efficacy and health outcomes for patients grappling with CCS.

## 1. Introduction

In 2019, the European Society of Cardiology (ESC) updated its guidelines for the diagnosis and management of chronic coronary syndrome (CCS), leading to a significant reclassification within coronary artery disease (CAD). The term “stable coronary artery disease” (SCAD) was replaced by “CCS,” and CAD was divided into acute coronary syndrome (ACS) and CCS. The guidelines characterize CAD as a chronic process [1, 2], acknowledging that atherosclerotic lesions within the coronary artery system are dynamic [3], span a wide spectrum, and exhibit a changing natural history, involving various parts of the coronary circulation [4]. Moreover, they incorporate stages of asymptomatic coronary heart disease, myocardial ischemia, vasospasm, and microcirculatory lesions, alongside the clinical manifestations predominantly marked by acute coronary thrombosis. This overhaul is rooted in the understanding that coronary heart disease is a dynamic process, marked by the accumulation of atherosclerotic plaque and changes in coronary circulation function, leading to both relatively stable periods and potential instability due to plaque rupture, plaque erosion, and calcified nodules, hence emphasizing the dynamic nature of coronary heart disease. In alignment with this, China's 2018 revised guidelines delineate the stable course following chronic stable labor angina, ischemic cardiomyopathy, and ACS as stable coronary heart disease, all of which share a common pathogenesis and pathophysiological basis. Consequently, patients with these conditions are collectively referred to as CCS patients [2]. Therefore, this systematic review includes guidelines that cover stable coronary artery disease, chronic coronary artery disease, chronic myocardial ischemia syndrome, stable angina, and exertional angina, even those published before 2019. CCS represents a growing clinical challenge worldwide [5], being a leading cause of morbidity and mortality [6], with an extremely high case fatality rate, significantly endangering public health and safety. Therefore, optimizing the management of CCS patients is of paramount importance.

Clinical practice guidelines (CPGs) are crucial in medicine, providing physicians and other healthcare professionals with evidence-based recommendations for the care of patients with various diseases or clinical conditions [7]. Although several CPGs for CCS have been issued, discrepancies exist between their recommendations. These variations might be due to the fact that CPGs from different countries are based on different sources and qualities of evidence. This review aims to summarize and compare the quality and consistency of CPGs for the diagnosis, treatment, and management of CCS patients, aiming to assist clinicians in making more informed clinical decisions and urging CCS guideline developers to thoroughly consider the quality of the evidence.

## 2. Methods

### 2.1. Search and Selection

**Figure 1:**
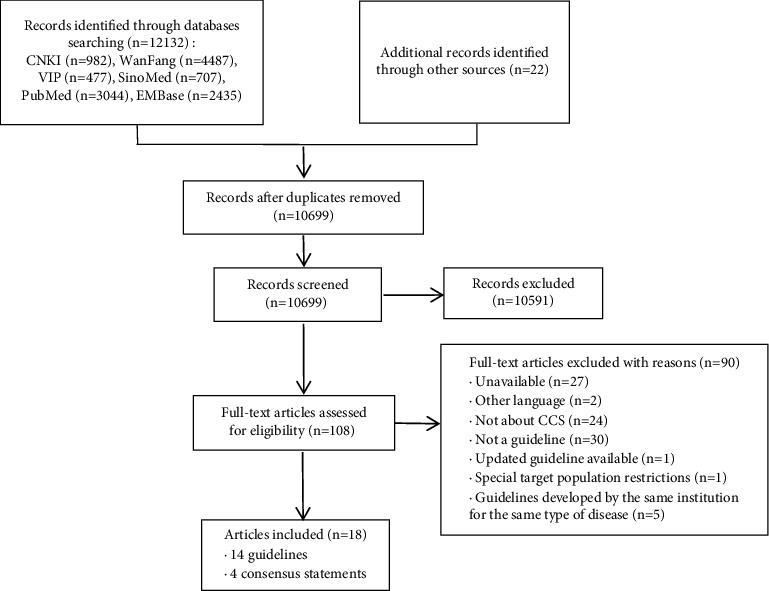
Screening chart of this study.

We conducted a comprehensive search across a range of databases including PubMed, EMBASE, China National Knowledge Infrastructure, Wanfang Database, China Science and Technology Journal Database, Chinese Biomedical Literature Service System, Chinese Science and Technology Periodical Database, and Chinese Biological Medicine Database, spanning from their respective inception dates until May 30, 2022. For our search, we utilized a variety of keywords, including “chronic coronary syndrome [Mesh],” “CCS [Title/Abstract],” “stable coronary artery disease [Title/Abstract],” “chronic coronary artery disease [Title/Abstract],” “chronic myocardial ischemia syndrome [Title/Abstract],” “Coronary Heart Disease [Title/Abstract],” “CAD [Title/Abstract],” “CHD [Title/Abstract],” “Angina, Stable [Title/Abstract],” “SAP [Title/Abstract],” “chronic angina [Title/Abstract],” “exertional angina [Title/Abstract],” “angina [Title/Abstract],” and “stable angina [Title/Abstract],” in conjunction with “Guideline [MeSH],” “guideline [Title/Abstract],” “expert consensus [Title/Abstract],” or “recommendation statement [Title/Abstract].” These keywords were appropriately adjusted and applied in Chinese databases using their equivalent terms in Chinese; the detailed search strategies are depicted in Figure 1.

In addition to database searches, we explored a selection of websites for relevant literature, including the National Group Standard Information Platform, Chinese Medical Association, Google Scholar, National Institute for Health and Care Excellence, Scottish Intercollegiate Guidelines Network, Guidelines International Network, World Health Organization, and the Cochrane Library. The task of selecting pertinent clinical practice guidelines and consensus statements was undertaken independently by two researchers.

### 2.2. Eligibility Criteria

CPGs were deemed eligible for inclusion based on the following criteria:

- Emphasis on the diagnosis, and/or treatment, and/or management of adult patients diagnosed with chronic coronary syndromes
- Provision of specific recommendations tailored to this patient demographic
- Absence of special target population restrictions, which encompasses but is not limited to pregnant women and elderly individuals
- Development under the auspices of nonprofit entities, including academic institutions, government agencies, disease-specific foundations, or professional associations or societies
- Publication in either Chinese or English, ensuring accessibility and comprehensibility
- Exclusion of documents classified as protocols, abstracts, editorial comments, overviews, review articles, and systematic reviews, to maintain a focus on comprehensive and authoritative sources
- Disqualification of multiple versions of CPGs developed by the same organization or group, with preference given to the most updated version
- In instances where CPGs were developed by the same institution for the same type of disease, preference was given to the version encompassing the most comprehensive content, thus ensuring a thorough and an extensive review

These criteria were meticulously crafted to ensure the inclusion of robust, relevant, and authoritative guidelines, facilitating a comprehensive analysis and comparison of the recommendations for the diagnosis, treatment, and management of chronic coronary syndromes.

### 2.3. Assessment of CPGs Quality

The Appraisal of Guidelines for Research and Evaluation II instrument (AGREE II) serves as a pivotal tool designed to tackle the issue of guideline quality variability, providing a rigorous and transparent framework for guideline development assessment. It encompasses 23 quality items, systematically distributed across six domains, alongside an additional item for overall quality assessment [8]. These domains include the following: (1) scope and purpose (items 1–3), (2) stakeholder involvement (items 4–6), (3) rigor of development (items 7–14), (4) clarity of presentation (items 15–17), (5) applicability (items 18–21), (6) editorial independence (items 22-23), and (7) an overall assessment (item 24). Each item within these categories is evaluated by using a seven-point scale, where a score of 1 denotes “strongly disagree” and a score of 7 implies “strongly agree.” To calculate the domain scores, expressed as a percentage of the maximum possible score for that specific domain, the following formula was utilized:

(1) $$\begin{array}{c} \text{domain score}=\left(\frac{\text{obtained score}-\text{minimum possible score}}{\text{maximum possible score}-\text{minimum possible score}}\right). \end{array}$$

The maximum possible score = the highest possible score (i.e., 7) × number of items × number of appraisers (i.e., 2). The minimum possible score = the lowest possible score (i.e., 1) × number of items × number of appraisers (i.e., 2).

Reflecting on the outcomes of prior published works [9, 10], we categorized the domain scores into three distinct tiers: high-quality (spanning 67–100%), sufficient quality (ranging from 33 to 67%), and low quality (encompassing 0–33%). The appraisal of CPGs' quality was independently conducted by two reviewers, both external to the guideline development group, by utilizing the AGREE II instrument. In instances where an AGREE II item is absent in a CPG, a score ranging from 0 to 3 is assigned, contingent on the specific context. If disagreements arise, a third reviewer intervenes, facilitating a resolution until a consensus is achieved.

### 2.4. Basic Characteristics of the Included Documents and Extraction of Recommendations

Two reviewers independently carried out the extraction of both basic characteristics and recommendation content from the included CPGs, covering areas such as (1) information pertaining to the development of the guideline (e.g., the development group, country, and year of publication), (2) the scope and content of the guideline (e.g., target population, diagnostic procedures, treatment options, and management strategies), (3) the grading systems used for the recommendations (e.g., the level of evidence and the strength of the recommendations), and (4) the specific recommendations put forth in the guidelines (e.g., for diagnosis and assessment, treatment, management, and rehabilitation).

## 3. Results

### 3.1. Selection of CPGs

The comprehensive search resulted in a total of 10,699 citations, ultimately yielding eighteen CPGs that provided specific recommendations. This ensemble comprised fourteen guidelines [11–24] and four consensus statements [12, 25–27]. A meticulously detailed flowchart, delineating the process of search and selection, is depicted in Figure 1.

Geographically, the development of these CPGs spanned across various regions: six CPGs [11–14, 25, 28] originated in China, four [16, 19, 22, 24] were formulated in Britain, two [17, 23] emerged from the United States, with Japan [20], Asia [26], Brazil [21], Malaysia [18], Italy [27], and Europe [15] each contributing one. Note that, one of the CPGs had undergone previous iterations and updates [20].

In terms of content, fifteen of the CPGs [11–15, 18–26, 28] incorporated recommendations pertinent to diagnosis and assessment. Recommendations regarding treatment were found in fifteen CPGs [11–22, 24, 26, 27], and management strategies were addressed in thirteen CPGs [11, 13–16, 18–22, 24, 25, 28]. The intricate characteristics and specific details of the eligible CPGs are meticulously cataloged and can be accessed from Supplementary Table 1.

### 3.2. Quality Assessment

The results of appraising the CPGs by using the AGREE II instrument are delineated in Supplementary Table 2. The evaluations yielded high-quality scores in three specific domains (domains 1, 2, and 4), whereas the remaining domains (domains 3, 5, and 6) achieved scores indicative of sufficient quality. The domain entitled “clarity of presentation” (domain 4) stood out with the highest average score of 92%, closely followed by the domain “scope and purpose” (domain 1) with an average score of 89%. On the other end of the spectrum, the domain “applicability” (domain 5) registered the lowest average score, coming in at 47%. In a general sense, the CPGs under review attained an average score that reflects sufficient quality, marked at 69%. Furthermore, both evaluators concurred in their recommendation of fourteen CPGs [11–16, 18–24, 26]. In contrast, the remaining four CPGs [17, 25, 27, 28] received a recommendation from at least one of the two evaluators, albeit with specified conditions for alterations.

### 3.3. Diagnosis and Assessment

Fifteen CPGs [11–15, 18–26, 28] have provided extensive coverage on the diagnosis and assessment of CCS. The recommendations within these CPGs predominantly focus on (in more than 50% of the guidelines) the assessment and stratification of risk factors, the evaluation of symptoms, and the utilization of ECG and ultrasonography. Among these, the assessment of risk factors stands out as the most frequently addressed recommendation. On the other hand, a smaller proportion of CPGs (in less than 50% of the guidelines) underscore the significance of applying invasive diagnostic methods, PE, X-rays, laboratory investigations, and stress testing. Note that, although both CTA [14, 15, 18–21, 26] and CAG [11, 14, 15, 19–21, 26] are endorsed in seven CPGs under invasive diagnosis, they find no recommendation in one particular CPG [13]. Comprehensive details and levels of recommendations pertinent to diagnosis and assessment are meticulously laid out in Supplementary Table 3.

### 3.4. Treatment

Fifteen CPGs [11–22, 24, 26, 27] provided recommendations regarding the treatment of CCS. It was noted that the treatment recommendations primarily fell into two categories: pharmacological and nonpharmacological, with pharmacotherapy being the predominant form of treatment. The pharmacologic interventions served two main therapeutic objectives: preventing and improving patient outcomes, and alleviating symptoms. Thirteen CPGs [13–22, 24, 26, 27] encompassed recommendations for the prevention and enhancement of outcomes, with aspirin being the most frequently recommended medication in these instances. In terms of drug treatments intended for symptom relief, the guidelines predominantly emphasized the utilization of beta-blockers, CCBs, and nitrates, as indicated by their recommendation in a majority of the guidelines (no. of guidelines ≥ 50%). Pertaining to nonpharmacological treatments, the prevalent recommendation in most CPGs (no. of guidelines ≥ 50%) was for PCI and CABG. It is crucial to highlight that two specific CPGs [16, 24] categorically contraindicated the application of TENS and EECP. Moreover, the recommendations regarding the employment of acupuncture varied across two CPGs [11, 24]. The comprehensive details and gradings of these treatment recommendations can be found in Supplementary Table 4.

### 3.5. Management and Rehabilitation

Thirteen CPGs [11, 13–16, 18–22, 24, 25, 28] offered comprehensive recommendations on the management and rehabilitation of CCS. Upon careful examination of these recommendations, we discerned that they spanned five key domains: management of risk factors, oversight during the rehabilitation period, reevaluation in cases of ineffective treatment, referrals for inconclusive treatments, and the imperative for a multidisciplinary approach involving various health professionals. The recommendations predominantly centered around (as indicated by coverage in over 50% of the guidelines) the management of risk factors, with exercise emerging as the most frequently endorsed recommendation.

Amongst the other CPGs, there was a pronounced emphasis on the cessation of smoking, along with the meticulous control of blood pressure and blood glucose levels, dietary management, and weight regulation. It is noteworthy to highlight that five CPGs [11, 13, 18, 20, 28] advocated for dietary therapy, while one particular CPG [24] abstained from doing so.

Pertaining to the domain of rehabilitation management, the guidelines collectively endorse regular reviews and assessments. A subset of these guidelines further recommends a holistic rehabilitation approach, encompassing nutritional guidance, exercise (including aerobic exercises, strength training, balance exercises, and flexibility training), and the potential integration of traditional Chinese or Western medicinal practices.

Moreover, there is a consistent call across various guidelines for the reassessment of treatment efficacy, urging referrals for cases deemed ineffective, and underscoring the value of a multidisciplinary approach that harnesses the expertise of diverse health professionals.

The intricate details and the stratification of these management recommendations are meticulously documented in Supplementary Table 5.

## 4. Discussion

This systematic review scrutinized a total of eighteen CPGs [11–28], all pertinent to the diagnosis, treatment, and management of CCS. A substantial variability was observed in the quality of these CPGs, particularly pronounced in domains such as applicability and editorial independence. In addition, there were notable discrepancies in the recommendations across these CPGs.

### 4.1. Quality of Current CPGs for CCS

Utilizing the AGREE II tool for assessment, it became evident that the majority of the CPGs exhibited commendable clarity concerning their scope and purpose. Nonetheless, there was a general inadequacy in the domains of the rigor of development, applicability, and editorial independence, with the domain of “applicability” scoring particularly low. For instance, only a selected few CPGs [13, 15, 16, 18–26] explicitly provided guidance or tools to facilitate the application of their recommendations. In contrast, the majority of the remaining CPGs [11, 12, 14, 15, 17, 27, 28] were found to be deficient, lacking in both implementation strategies and the requisite resources to effectively put the recommended practices into action. Although several CPGs [11–13, 16, 18, 19, 22, 26] claimed to employ systematic methodologies in evidence retrieval, there were others [14, 15, 17, 20, 21, 23–25, 27, 28] that did not, signaling a need for heightened emphasis on the rigor of evidence in future iterations of CPGs. Furthermore, a considerable number of CPGs [13, 14, 17, 20–23, 25–28] fell short of mentioning the procedures for guideline updates, with only a small fraction [11, 12, 15, 16, 18, 19, 24] addressing this aspect, a practice that should be made mandatory in forthcoming guideline development endeavors.

In summation, the quality of these CPGs was found to be extensively variable. The variability observed across different domains could be attributed to the diverse nature of the development bodies, and/or the potential lack of adherence to a standardized set of criteria during their formulation.

### 4.2. Key Findings

In this review, we incorporated a total of eighteen CPGs pertinent to CCS and proceeded to analyze the consistency across the recommendations. However, we identified some degree of variation amongst these CPGs.

Divergences are noticeable among fifteen CPGs [11–15, 18–26, 28], specifically in terms of the recommendations for the diagnosis and assessment of CCS. For instance, CTA is advocated for diagnosing or assessing the risk of CCS in seven guidance documents [14, 15, 18–21, 26], while another CPG [13] holds a contrary stance. It articulates that routine utilization of CTA for the diagnosis or risk stratification of coronary heart disease is not advisable. Nonetheless, CTA may be considered if there are contraindications to stress testing, or when functional tests are inconclusive in determining the diagnosis or risk level. The same CPG [13] also dissuades routine CAG testing in patients exhibiting normal left ventricular systolic function, low risk upon noninvasive testing, and absence of ischemia on asymptomatic, noninvasive testing. Conversely, seven CPGs [11, 14, 15, 19–21, 26] endorse the use of CAG for the diagnosis or risk assessment of CCS. Hence, the question of whether CTA and CAG tests should be routinely recommended for the diagnosis and evaluation of CCS, along with their specific clinical applications, awaits consensus and resolution in future CPGs.

Discrepancies are also observed in fifteen CPGs [11–22, 24, 26, 27] that furnish recommendations for treating CCS. In the context of nonpharmacologic treatments, one CPG [11] recommends acupuncture as a means to alleviate CCS symptoms and ameliorate myocardial ischemia, while another [24] explicitly discourages its use for CCS patients. Despite its widespread clinical application in China, acupuncture remains a contentious method for treating chronic pain, partly due to the fact that traditional acupuncture theory, with its emphasis on meridians and nonphysiological processes such as qi energy flow, is not readily explicable through anatomical terms. A comprehensive meta-analysis [29], encompassing 39 trials with a total of 20,827 patients, concluded that acupuncture is effective for chronic pain, and its benefits persist over time. Some studies [30–32] posit that acupuncture can exert short-term physiological effects related to pain relief. However, the data required to elucidate the persistence of this effect and the mechanisms through which acupuncture mitigates pain are still deficient [29]. It is our hope that future CPGs can address this controversy, and we underscore the need for more extensive, multicenter, high-quality clinical studies to substantiate the clinical efficacy of acupuncture in treating chronic pain.

Among the recommendations pertaining to the management and rehabilitation of CCS, five guidelines [11, 13, 18, 20, 28] advocate for the utilization of diet therapy in addressing CCS. Conversely, there is one guideline [24] that takes a differing stance, advising against this approach. It articulates a clear position, stating that there is a lack of compelling evidence to support the use of vitamin or fish oil supplements as a treatment for stable angina. Furthermore, it highlights the absence of research exploring the impact of dietary modifications on both morbidity and mortality rates. Consequently, it underscores the necessity for randomized controlled trials to undertake a comparative analysis between comprehensive cardiac rehabilitation and standard care for patients diagnosed with stable angina [24].

On a broader scale, there is a noticeable degree of variability across the existing CPGs pertaining to the diagnosis, treatment, and management of CCS. This variability can, in part, be attributed to the fact that CPGs, originating from different organizations or geographical regions, are grounded in diverse resources and possess varying levels of evidence quality.

In addition, CCS has traditionally been conceptualized as a consequence of induced ischemia. Observing the treatment recommendations put forth by the existing CPGs, it becomes apparent that the primary focus of treatment is centered around the reduction or elimination of induced ischemia. This is predominantly achieved through pharmacological interventions such as beta-blockers, CCBs, nitrates, and strategies such as interventional therapy or bypass surgery. However, recent data accumulation suggests that the atherosclerotic load plays a more pivotal role in determining the outcomes of CCS, specifically in terms of the quantity and stability of coronary plaque. This holds true even in scenarios where induced ischemia is not prominently featured. The existence and overall load of atherosclerosis serve to elevate the risk of cardiovascular events. In this context, the ISCHEMIA [33], REVIVED-BCIS2 [34], and ORBITA [35] clinical trials have emerged as significant and representative studies within the realm of CCS in recent years. The outcomes of these trials collectively highlight that optimal pharmacotherapy and conservative treatment should be prioritized as the primary course of action for CCS patients. In addition, they call for more stringent criteria to be applied when considering coronary stent therapy. These findings offer valuable insights for CCS patients, illuminating the trade-offs between invasive and conservative treatments. They also validate the notion that an exclusive focus on the reduction or elimination of induced ischemia through interventional means does not yield satisfactory outcomes. Consequently, there is a pressing need to reorient the treatment objectives for CCS, shifting the focus towards the reduction of arterial plaque and alleviating the atherosclerotic burden. Treatment strategies should pivot towards arresting the progression of atherosclerosis, incorporating more potent antiplatelet drugs, anticoagulant medications, and agents that foster plaque stabilization. This approach not only reduces plaque formation and progression but also alleviates symptoms in CCS patients, subsequently mitigating the risk of cardiovascular events. The existing CPGs exhibit a near-total omission of evidence stemming from clinical evidence-based studies such as ISCHEMIA, REVIVED-BCIS2, and ORBITA. This oversight has the potential to impinge on the precision of clinicians' treatment decisions for CCS patients, potentially depriving patients of the most efficacious treatment options.

### 4.3. Strengths and Limitations

This investigation stands as a pioneering endeavor to systematically review the quality of CPGs pertinent to CCS. In the process of this study, a thorough and systematic approach to literature search was employed with the aim of identifying CPGs that are relevant to the diagnosis, treatment, and management of CCS. As for the content within these CPGs, we opted for the utilization of charts as a means to provide a visual comparison of the consistency and disparities present in the recommendations across different CPGs. In addition, we took the initiative to highlight the strength of recommendations in certain guidelines, thereby enabling the target demographic to glean high-frequency or high-intensity recommendations in a more intuitive manner. The evaluation of the CPGs was conducted independently by two reviewers, employing the AGREE II tool, which subsequently revealed a significant degree of variability within the published CPGs for CCS. This was particularly evident in the domains of applicability and editorial independence. The insights gained from this could potentially serve as a valuable resource for the enhancement of future CCS guidelines.

Nevertheless, it is imperative to acknowledge the limitations that accompany this study. The decision to exclude CPGs that were not written in Chinese or English may have resulted in the inadvertent omission of certain pertinent CPGs. Consequently, this may render the conclusions drawn from this study somewhat constrained in their applicability to other countries or regions, due to the language restrictions in place. Moreover, it is worth noting that AGREE II, the tool employed for domain score evaluation, lacks universally recognized cutoff points. In light of this, our approach involved referring to prior articles [9, 10] that addressed domain scores, a decision that is not without its potential for controversy.

## 5. Conclusions and Future Recommendations

The introduction of CCS as a concept not only heralds a shift in nomenclature but also signifies an enhancement in clinical researchers' grasp of the pathogenesis underlying CAD. This paradigmatic shift draws attention to the significance of atherosclerotic burden, moving beyond merely focusing on inducible ischemia, a nuance that has been insufficiently captured in existing CPGs. There is a noticeable divergence in both the quality and the recommendations emanating from the published guidelines specific to CCS, underscoring an imperative for interdisciplinary collaboration to forge comprehensive and evidence-based CPGs. Such guidelines are pivotal in championing optimal treatment pathways and improving health outcomes for individuals grappling with CCS.

Furthermore, it is advocated that the development of CPGs adheres to standardized protocols, exemplified by the AGREE II evaluation criteria. This approach is instrumental in pinpointing and bridging the extant gaps within CPGs, thereby furnishing users with a more holistic suite of information. Although the majority of CPGs find their roots in systematic literature reviews, discrepancies remain prevalent, particularly concerning the criteria employed to categorize evidence and the methodologies utilized to formulate recommendations. These discrepancies inevitably lead to variations in the level of recommendation accorded to identical interventions across different CPGs. There is, therefore, a pressing need to standardize the procedures and methodologies that underpin the formation of recommendation opinions, aiming to enhance both the consistency and usability of the guidelines.

Moreover, it is of paramount importance to take into account diverse subgroups and clinical settings in the development of CPGs, recognizing that they may harbor unique needs and prerequisites. Recommendations that are meticulously tailored to these specific subgroups and settings stand to augment clinical efficacy and simplify the utilization of the guidelines for healthcare professionals. In addition, a comprehensive literature search revealed a scarcity of clinical and epidemiological studies focusing on CCS, highlighting a promising avenue for future research endeavors in this domain.

## Data Availability

The primary data for this study were guidelines, which are available on request from the corresponding authors.

## Supplementary Materials

Supplementary Materials The comprehensive details of intricate characteristics, quality assessment results, and recommendations pertinent to the diagnosis, treatment, and management of the eligible CPGs can be accessed in Supplementary Materials.
