# Supplementary material for: Diagnosis, Treatment, and Management for Chronic Coronary Syndrome: A Systematic Review of Clinical Practice Guidelines and Consensus Statements
Source: Int J Clin Pract. 2023 Dec 18;2023:9504108. doi: 10.1155/2023/9504108 (PMC10749717; doi:10.1155/2023/9504108)
Supplement: Supplementary Materials — The comprehensive details of intricate characteristics, quality assessment results, and recommendations pertinent to the diagnosis, treatment, and management of the eligible CPGs can be accessed in Supplementary Materials. [file 9504108.f1.zip › Supplementary Table 1.docx]

**Supplementary Table 1**| Guidelines and consensus statement included in this paper.

| **Guideline** | **Institution/Group** | **Country** | **Year** | **Target population** | **diagnosis and assessment** | **Treatment** | **Management and rehabilitation** | **Grading of**  **evidence** |
| --- | --- | --- | --- | --- | --- | --- | --- | --- |
| Wang et al. [11] | CACM | China | 2019 | CAD/ SA | Yes | Yes | Yes | Yes |
| Li et al. [12] | CRACM | China | 2019 | SCAD | Yes | No | Yes | No |
| Mao et al. [13] | Standardization project group of Clinical Application Guide for Dominant Diseases of Chinese Patent Medicine Treatment | China | 2020 | CAD | Yes | Yes | No | Yes |
| Han et al. [14] | National Health and Family Planning Commission of the People's Republic of China, CPA | China | 2018 | CAD | Yes | Yes | Yes | Yes |
| Mi et al. [15] | CMDA | China | 2021 | CCS | Yes | No | Yes | No |
| Fu et al. [16] | CSC, CMDA, Editorial Board of Chin J Cardio | China | 2018 | SCAD | Yes | Yes | Yes | Yes |
| Knuuti et al. [17] | ESC | European | 2019 | CCS | Yes | Yes | Yes | Yes |
| Jack Tan et al. [18] | APSC | Asian | 2020 | CCS | Yes | Yes | No | Yes |
| Timmis et al. [19] | NCGC | Britain | 2011 | SA | No | Yes | Yes | Yes |
| Snow et al. [20] | ACC/AHA | American | 2004 | CSA | No | Yes | No | Yes |
| Rajadurai et al. [21] | NHAM, AMM | Malaysia | 2018 | SCAD | Yes | Yes | Yes | Yes |
| Bennison et al. [22] | SIGN | Britain | 2018 | SA | Yes | Yes | Yes | Yes |
| Nakano et al. [23] | JCS | Japan | 2022 | SCAD | Yes | Yes | Yes | Yes |
| Cesar et al. [24] | BSA | Brazil | 2014 | SCAD | Yes | Yes | Yes | Yes |
| Ferrari et al. [25] | University of Ferrara | Italy | 2017 | CSA | No | Yes | No | No |
| Martin et al. [26] | The North of England SA Guideline Development Group | Britain | 2001 | SA | Yes | Yes | Yes | Yes |
| Snow et al. [27] | ACC/AHA | American | 2004 | CSA | Yes | No | No | Yes |
| Timmis et al. [28] | NICE | Britain | 2011 | SA | Yes | Yes | Yes | No |

CACM, China Association of Chinese Medicine; CAD, Coronary artery disease; SA, Stable angina; CRACM, Chinese Association for Research and Advancement of Chinese Medicine; SCAD, Stable coronary artery disease; CPA, Chinese Pharmacists Association; CMDA, Chinese Medical Doctor Association; CCS, Chronic coronary syndrome; CSC, Chinese Society of Cardiology; Chin J Cardio, Chinese Journal of Cardiology; ESC, The European Society of Cardiology; APSC, The Asian Pacific Society of Cardiology; NCGC, The National Clinical Guidelines Centre; ACC, American College of Cardiology; AHA, American Heart Association; CSA, Chronic stable angina; NHAM, The National Heart Association of Malaysia; AMM, Academy of Medicine of Malaysia; SIGN, Scottish Intercollegiate Guidelines Network; JCS, Japanese Circulation Society; BSA, Brazilian Society of Cardiology; NICE, National institute for Health and Care Excellence; Yes, include relevant content in guidelines; No, no relevant content in guidelines; N/A, not applicable.
