# Supplementary material for: Diagnosis, Treatment, and Management for Chronic Coronary Syndrome: A Systematic Review of Clinical Practice Guidelines and Consensus Statements
Source: Int J Clin Pract. 2023 Dec 18;2023:9504108. doi: 10.1155/2023/9504108 (PMC10749717; doi:10.1155/2023/9504108)
Supplement: Supplementary Materials — The comprehensive details of intricate characteristics, quality assessment results, and recommendations pertinent to the diagnosis, treatment, and management of the eligible CPGs can be accessed in Supplementary Materials. [file 9504108.f1.zip › Supplementary Table 2.docx]

**Supplementary Table 2** | Assessment of guidelines and consensus statements by AGREE Ⅱ

| **Domains** | **No of guidelines** | | | **Average Score (%)** |
| --- | --- | --- | --- | --- |
|  | **Score 0-33%** | **Score 34–66%** | **Score 67–100%** |  |
| Scope and purpose | 0 | 1 | 17 | 89 |
| Stakeholder involvement | 0 | 9 | 9 | 67 |
| Rigor of development | 2 | 9 | 7 | 62 |
| Clarity of presentation | 0 | 1 | 17 | 92 |
| Applicability | 8 | 5 | 5 | 47 |
| Editorial independence | 5 | 5 | 8 | 57 |
| Overall | 0 | 6 | 12 | 69 |

0–33%, low quality; 34–66%, sufficient quality; 67–100%, high quality.
