# Supplementary material for: Diagnosis, Treatment, and Management for Chronic Coronary Syndrome: A Systematic Review of Clinical Practice Guidelines and Consensus Statements
Source: Int J Clin Pract. 2023 Dec 18;2023:9504108. doi: 10.1155/2023/9504108 (PMC10749717; doi:10.1155/2023/9504108)
Supplement: Supplementary Materials — The comprehensive details of intricate characteristics, quality assessment results, and recommendations pertinent to the diagnosis, treatment, and management of the eligible CPGs can be accessed in Supplementary Materials. [file 9504108.f1.zip › Supplementary Table 3.docx]

**Supplementary Table 3 |** Summary of recommendations for diagnosis and assessment

| **Recommendations** | **No of guidelines (%)** | **Recommended strength** | | | |
| --- | --- | --- | --- | --- | --- |
|  |  | **A** | **B** | **C** | **D** |
| Assessment of the risk factors | 12 (67%) | 2 | - | - | - |
| Symptoms | 10 (56%) | 4 | - | - | - |
| ECG | 10 (56%) | 5 | 1 | - | - |
| Ultrasonography | 9 (50%) | 4 | 1 | - | - |
| Risk stratification | 9 (50%) | 3 | - | - | - |
| Invasive diagnosis | 8 (44%) | 1 | - | - | - |
| CTA | 8 (44%) | 3 | 1 | - | 1 |
| CAG | 8 (44%) | 3 | 1 | - | 1 |
| FFR | 3 (38%) | 1 | 1 | - | - |
| ICA | 3 (38%) | 2 | - | - | - |
| PE | 7 (39%) | 3 | - | - | - |
| X-Ray | 7 (39%) | 3 | 2 | - | - |
| Laboratory investigations | 6 (33%) | 4 | - | - | - |
| Load check | 6 (33%) | 3 | 1 | - | - |
| CMR | 5 (28%) | 2 | - | - | - |
| MPI | 5 (28%) | 1 | 1 | - | - |
| Clinical history collection | 5 (28%) | 1 | - | - | - |
| CCTA | 4 (22%) | 2 | - | - | - |
| CS | 4 (22%) | 1 | 1 | 1 | - |
| For different PTP level group, select different non-invasive imaging techniques | 3 (38%) | - | - | - | - |
| ETT | 2 (11%) | 1 | - | - | - |
| Provide long-term dynamic evaluation | 1 (6%) | - | - | - | - |

ECG, Electrocardiogram; CTA, Coronary artery imaging; CAG, Coronary angiography; FFR, Measurement of Blood Flow reserve grade; ICA, Invasive coronary angiography; PE, physical examination; CMR, Cardiac Magnetic Resonance Imaging; MPI, Myocardial perfusion imaging; CCTA, Non-invasive anatomical; CS, The calcium score; ETT, Exercise treadmill test; A, strong recommendation; B, moderate recommendation; C, week recommendation; D, not recommended; -, not applicable. Guidelines that do not mention recommendations for diagnosis and assessment are not shown in the table, and recommended intensities involve extraction at the highest recommended intensity for multiple clinical situations.
