# Supplementary material for: Diagnosis, Treatment, and Management for Chronic Coronary Syndrome: A Systematic Review of Clinical Practice Guidelines and Consensus Statements
Source: Int J Clin Pract. 2023 Dec 18;2023:9504108. doi: 10.1155/2023/9504108 (PMC10749717; doi:10.1155/2023/9504108)
Supplement: Supplementary Materials — The comprehensive details of intricate characteristics, quality assessment results, and recommendations pertinent to the diagnosis, treatment, and management of the eligible CPGs can be accessed in Supplementary Materials. [file 9504108.f1.zip › Supplementary Table 4.docx]

**Supplementary Table 4 |** Summary of recommendations for treatment

| **Recommendations** | **No of guidelines (%)** | **Recommended strength** | | | |
| --- | --- | --- | --- | --- | --- |
|  |  | **A** | **B** | **C** | **D** |
| Pharmacologic treatments | 15 (83%) | - | - | - | - |
| Prevent and improve outcomes | 13 (72%) | - | - | - | - |
| Aspirin | 12 (67%) | 5 | - | - | - |
| Clopidogrel | 9 (50%) | 4 | 1 | - | - |
| ACEI-ARB | 8 (44%) | 4 | - | - | - |
| Statins | 7 (39%) | 3 | - | - | - |
| Beta blockers | 5 (28%) | 3 | - | - | - |
| Nitrates | 4 (22%) | 1 | - | - | - |
| OAC | 4 (22%) | 3 | - | - | - |
| CCBs | 3 (38%) | 1 | - | - | - |
| Ticagrelor | 2 (11%) | - | - | 1 | - |
| Ticlopidine | 2 (11%) | - | 1 | 1 | - |
| Triflusal | 1 (6%) | - | - | 1 | - |
| Ivabradine | 1 (6%) | - | - | - | - |
| rhBNP | 1 (6%) | - | - | - | - |
| Fibrates | 1 (6%) | - | - | - | - |
| Ezetimibe | 1 (6%) | - | - | - | - |
| Resins | 1 (6%) | - | - | - | - |
| Niacin | 1 (6%) | - | - | - | - |
| Relieve symptoms | 14 (78%) | - | - | - | - |
| Beta blockers | 12 (67%) | 5 | 1 | - | - |
| CCBs | 12 (67%) | 3 | 2 | - | - |
| Nitrates | 12 (67%) | 5 | - | - | - |
| Ivabradine | 9 (50%) | - | 3 | - | - |
| Ranolazine | 9 (50%) | - | 1 | - | - |
| Nicorandil | 9 (50%) | 1 | 1 | - | - |
| Combination therapy | 9 (50%) | 2 | - | - | - |
| Trimetazidine | 5 (28%) | - | 2 | 1 | - |
| Chinese traditional patent medicine | 3 (38%) | 1 | - | - | - |
| Chinese herbal decoction | 1 (6%) | - | - | - | - |
| Non-pharmacological treatments | 11 (61%) | - | - | - | - |
| PCI | 10 (56%) | 4 | - | - | - |
| CABG | 9 (50%) | 4 | - | - | - |
| TENS | 2 (11%) | - | - | - | 2 |
| EECP | 2 (11%) | - | - | - | 2 |
| Acupuncture | 2 (11%) | - | - | - | 1 |
| Acupoint application | 1 (6%) | - | - | - | - |
| Massage | 1 (6%) | - | - | - | - |
| Traditional exercise of TCM | 1 (6%) | - | - | - | - |

ACEI/ARB, angiotensin converting enzyme inhibitor/angiotensin receptor blocker; OAC, anticoagulant; CCBs, calcium channel blockers; rhBNP, Natriuretic peptides; PCI, percutaneous coronary intervention; CABG, coronary artery bypass grafting; TENS, transcutaneous electrical nerve stimulation; EECP, Enhanced External Counter pulsation; A, strong recommendation; B, moderate recommendation; C, week recommendation; D, not recommended; -, not applicable. Guidelines that do not mention recommendations for treatment are not shown in the table, and recommended intensities involve extraction at the highest recommended intensity for multiple clinical situations.
