# Supplementary material for: Diagnosis, Treatment, and Management for Chronic Coronary Syndrome: A Systematic Review of Clinical Practice Guidelines and Consensus Statements
Source: Int J Clin Pract. 2023 Dec 18;2023:9504108. doi: 10.1155/2023/9504108 (PMC10749717; doi:10.1155/2023/9504108)
Supplement: Supplementary Materials — The comprehensive details of intricate characteristics, quality assessment results, and recommendations pertinent to the diagnosis, treatment, and management of the eligible CPGs can be accessed in Supplementary Materials. [file 9504108.f1.zip › Supplementary Table 5.docx]

**Supplementary Table 5 |** Summary of recommendations for management and rehabilitation

| **Recommendations** | **No of guidelines (%)** | **Recommended strength** | | | |
| --- | --- | --- | --- | --- | --- |
|  |  | **A** | **B** | **C** | **D** |
| Management of risk factors | 11 (61%) | 2 | - | - | - |
| Exercises | 9 (50%) | 3 | 1 | - | - |
| Quit smoking | 7 (39%) | 4 | - | - | - |
| Blood lipids | 7 (39%) | 3 | - | - | - |
| Blood pressure | 7 (39%) | 3 | - | - | - |
| Blood glucose | 6 (33%) | - | 1 | - | - |
| Diet therapy | 6 (33%) | 1 | - | - | 1 |
| Control weight | 5 (28%) | 2 | 1 | - | - |
| Limit alcohol consumption | 4 (22%) | 2 | - | - | - |
| Social and psychological | 4 (22%) | 1 | 1 | - | - |
| Patient education | 4 (22%) | 2 | - | - | - |
| Rehabilitation period management | 4 (22%) | 1 | - | - | - |
| Review regularly | 4 (22%) | 1 | - | 1 | - |
| Psychological rehabilitation | 4 (22%) | 1 | - | - | - |
| Exercise rehabilitation | 4 (22%) | 1 | - | - | - |
| Aerobic exercise | 1 (6%) | - | - | - | - |
| Strength training | 1 (6%) | - | - | - | - |
| Flexibility training | 1 (6%) | - | - | - | - |
| Nutritional rehabilitation | 3 (38%) | 1 | - | - | - |
| Rehabilitation assessment | 3 (38%) | 1 | - | - | - |
| Western medicine treatment | 3 (38%) | 1 | - | - | - |
| Chinese traditional treatment | 3 (38%) | 1 | - | - | - |
| Re-evaluation of treatment ineffectiveness | 4 (22%) | 1 | - | - | - |
| Inconclusive treatment should be referred | 3 (38%) | 1 | - | 1 | - |
| Multidisciplinary involvement of health professionals | 1 (6%) | 1 | - | - | - |

A, strong recommendation; B, moderate recommendation; C, week recommendation; D, not recommended; -, not applicable. Guidelines that do not mention recommendations for management and rehabilitation are not shown in the table, and recommended intensities involve extraction at the highest recommended intensity for multiple clinical situations.
